# Acceptability of Contraceptive Services in the Emergency Department: A Cross-sectional Survey

**DOI:** 10.5811/westjem.2021.2.49675

**Published:** 2021-05-24

**Authors:** Andreia B. Alexander, Kimberly Chernoby, Nathan VanderVinne, Yancy Doos, Navneet Kaur, Caitlin Bernard, Jeffrey A. Kline

**Affiliations:** * Indiana University School of Medicine, Department of Emergency Medicine, Indianapolis, Indiana; † Indiana University-Purdue University, School of Science, Indianapolis, Indiana; ‡ Indiana University School of Medicine, Department of Obstetrics and Gynecology, Indianapolis, Indiana

## Abstract

**Introduction:**

Unintended pregnancy disproportionately affects marginalized populations and has significant negative health and financial impacts on women, their families, and society. The emergency department (ED) is a promising alternative setting to increase access to sexual and reproductive health (SRH) services including contraception, especially among marginalized populations. The primary objective of this study was to determine the extent to which adult women of childbearing age who present to the ED would be receptive to receiving contraception and/or information about contraception in the ED. As a secondary objective, we sought to identify the barriers faced in attempting to obtain SRH care in the past.

**Methods:**

We conducted a quantitative, cross-sectional, assisted, in-person survey of women aged 18–50 in the ED setting at two large, urban, academic EDs between June 2018–September 2019. The survey was approved by the institutional review board. Survey items included demographics, interest in contraception initiation and/or receiving information about contraception in the ED, desire to conceive, prior SRH care utilization, and barriers to SRH.

**Results:**

A total of 505 patients participated in the survey. Participants were predominantly single and Black, with a mean age of 31 years, and reporting not wanting to become pregnant in the next year. Of those participants, 55.2% (n = 279) stated they would be interested in receiving information about birth control AND receiving birth control in the ED if it were available. Of those who reported the ability to get pregnant, and not desiring pregnancy in the next year (n = 279, 55.2%), 32.6% were not currently using anything to prevent pregnancy (n = 91). Only 10.5% of participants stated they had experienced barriers to SRH care in the past (n = 53). Participants who experienced barriers to SRH reported higher interest in receiving information and birth control in the ED (74%, n = 39) compared to those who had not experienced barriers (53%, n = 240); (P = 0.004, 95% confidence interval, 1.30–4.66).

**Conclusion:**

The majority of women of childbearing age indicated the desire to access contraception services in the ED setting. This finding suggests favorable patient acceptability for an implementation study of contraception services in emergency care.

## BACKGROUND

Despite the decline in unintended pregnancy rates in the United States over the past decade, unintended pregnancy remains a significant public health issue.^1^ According to the US Centers for Disease Control and Prevention (CDC), factors for increased risk of unintended pregnancy include the following: age 18–24 years; non-Hispanic Black; low income (<100% federal poverty level); less than high school education; and cohabitation without marriage.^2^,^3^ Additionally, unintended pregnancy has significant negative health and financial impacts on women, their families, and society.^4^–^8^

The decrease in unintended pregnancy rates in the US has been attributed to increased access and utilization of contraception.^2^ This decrease can largely be attributed to the contraception benefit of the Affordable Care Act (ACA), which required insurance companies to cover contraception without a copay.^9^ After implementation of the ACA we saw significant increases in contraception utilization and decreases in pregnancy rates, particularly in patients at highest risk for unintended pregnancy.^10^ However, with nearly three million unintended pregnancies per year,^11^ the US ranks significantly higher than many other developed countries.^12^ Thus, there is still significant room for improvement.

The emergency department (ED) is a promising alternative setting to increase access to sexual and reproductive health (SRH) services including contraception, especially among marginalized populations.^13^–^18^ Emerging evidence has suggested it is feasible to provide SRH services in the ED.^19^

A mandatory aspect of translating medical services from theory into practice (so-called implementation science, or T2 to T3 translation) requires input from patients. Given the dearth of literature on the role of SRH interventions in the ED setting, we conducted a cross-sectional survey to assess patients’ receptiveness to accepting contraception services in the ED. Survey studies are useful when trying to understand respondents’ opinions,^20^ such as in acceptability studies. The primary objective of this study was to determine the extent to which adult women of childbearing age who present to the ED would be receptive to receiving contraception and/or information about contraception in the ED. As a secondary objective, we sought to identify the barriers faced in attempting to obtain SRH care in the past.

## METHODS

### Study Design

We conducted a quantitative, cross-sectional, assisted, in-person survey of women aged 18–50 in the ED setting. This study was approved by the institutional review board at our institution.

### Participant Recruitment and Data Collection

A convenience sample of participants were recruited from two large, urban, academic EDs between June 2018–September 2019. Each ED has approximately 100,000 annual visits and serves primarily adult patients. (About 85% of visits at each site are by patients at least 18 years of age.) Eligible participants were women aged 18–50 who presented to the ED for any complaint when a research assistant (RA) was present in the ED. The RAs were volunteers and did not have a set schedule. While it was feasible to collect data 24 hours per day/seven days per week, the RAs dictated their own schedules. We excluded participants from the study if they were intoxicated, exhibiting hostile behavior, non-English speaking, or had a chief complaint of sexual assault (due to the potential introduction of psychological risk). Participants were approached and asked to participate in the study by a RA, after the RA confirmed appropriate timing with the treating emergency physician or resident. If they agreed, participants were given a study information sheet, questions were answered, and verbal consent was obtained. The RAs then verbally administered the survey to participants, capturing their responses electronically. The survey took approximately 10 minutes to complete. No compensation was provided for participation.

**Population Health Research Capsule**

What do we already know about this issue?

*Unintended pregnancy disproportionately affects marginalized populations and has significant negative health and financial impacts.*

What was the research question?

*Our goal was to determine whether women presenting to the emergency department (ED) would be receptive to contraceptive services in the ED.*

What was the major finding of the study?

*Most women were interested in accessing contraception in the ED setting.*

How does this improve population health?

*Increasing access to contraception in the ED for patients at higher risk for unintended pregnancy could help decrease this health inequity.*

### Survey Development

Survey items were developed by an EM resident (NV). To establish face and content validity, a multidisciplinary team of content experts from emergency medicine, obstetrics and gynecology, and pediatric-adolescent medicine, evaluated the initial survey items. Sequential changes were made to the instrument based on their discussions. Once the survey design was complete, the survey was pilot-tested on five lay family members of EM residents using a cognitive interviewing technique^21^ to identify issues with timing, wording, and skip patterns. We used feedback from these sessions to revise the survey. Once approved by the research team, the survey was ready for dissemination. The survey was transferred to an electronic data capture system (REDCap, Vanderbilt University, Nashville, TN); the complete survey is provided in the supplemental appendix.

### Measures

#### Demographics

Demographic questions included race, ethnicity, education, employment status, student status, and relationship status.

#### Acceptability

Acceptability of receiving contraception and/or information about contraception in the ED was measured by a single, multiple-choice question, “Would you be interested in receiving information about birth control or getting birth control in the ED if it was available?” Participants were given five choices: 1) yes, receive birth control and information; 2) yes, receive information only; 3) no; 4) unsure; and 5) other. To get a better understanding of the context of the participants’ answers we asked additional questions around the participants’ current desire/ability to become pregnant and current contraceptive choices. Examples of these questions include the following: “Would you like to become pregnant in the next year?” with the options of 1) yes, 2) no, and 3) unsure; and “Are you currently using anything to prevent pregnancy?” with the options of 1) intrauterine device (IUD), 2) contraceptive implant, 3) injectable birth control, 4) birth control pills, 5) patch, 6) vaginal ring, 7) condoms, 8) withdrawal, 9) natural family planning, 10) abstinence, and 11) other.

#### Sexual and reproductive health care

Where participants sought SRH care was determined by a single, multiple-choice question, “Where do you currently seek care for things like birth control, STIs, pap smears, or other GYN health issues?” Participants were given nine response items, with the option to choose more than one item: primary care physician; gynecologist; each ED used in this study listed separately; other ED, Planned Parenthood; institution-affiliated outpatient clinic; other outpatient clinic; and nowhere.

#### Barriers

We assessed barriers with two multiple-choice questions; the first question was “Have you had any difficulty getting care for things like birth control, STIs, pap smears, or other GYN health issues?” Participants were given yes/no response options. This question was followed up with, “What difficulties have you had?” Participants were given seven options, with the choice to select more than one option: difficulty finding a clinic; difficulty making an appointment; difficulty getting to an appointment; difficulty affording the visit; difficulty affording birth control, medications, etc; receiving criticism or judgment from clinic/staff/doctors/etc; and other.

#### Sample size and data analysis

A target sample size of 500 participants was determined to represent our ED population with a 95% confidence interval and 5% margin of error. We analyed data with SPSS Statistics 26 (IBM Corporation, Armonk, NY) using descriptive statistics and chi-squared analyses. Due to the nature of our data collection methods, there was <1% missing data.

## RESULTS

A total of 505 patients participated in the survey. Participants were predominantly single (n = 276; 54.7%) and Black (n = 240; 47.5%) with a mean age of 31 years (Table). Most (n = 471, 93%) of our participants were sexually active and the majority (n =2 79, 55.2%) also reported not wanting to become pregnant in the next year. Only 7.2% (n = 36) of participants reported primarily using the ED for SRH care needs, with an additional 12.3% (n = 62) of participants stating they did not go anywhere to seek SRH care.

Overall, 55.2% of participants (n = 279) stated they would be interested in receiving information about birth control AND receiving birth control in the ED if it were available. Another 7.3% (n = 37) reported wanting information only.

Of participants who self-reported having the ability to get pregnant (n = 382, 75.6%), 56.3% (n = 215) were currently using contraception. Participants were most likely to report using only condoms (n = 40; 10.4%), followed by abstinence (n = 36, 9.4%). Only 23.3% (n = 89) were using a form of long-acting reversible contraception (LARC): IUD (n = 34, 8.9%); implant (n = 30, 7.9%); or injectable (n = 25, 6.5%). The Figure reports a complete account of contraceptive use in participants with the ability to get pregnant.

Of the participants who reported the ability to get pregnant, and also not desiring pregnancy in the next year (n = 279, 55.2%), 32.6% were not using anything to prevent pregnancy at the time of the survey (n = 91). Furthermore, an additional 20.4% (n = 57) were using only condoms and 6.1% (n = 17) were using only the withdrawal method to prevent pregnancy. Similar to the overall sample, 56.6% of these participants stated they would be interested in receiving information about birth control and starting or changing their contraceptive method in the ED if it were available (n = 158).

When asked about barriers to obtaining SRH care, only 10.5% of participants stated they had experienced barriers to care (n = 53). The most common stated barriers to SRH were the following (in descending order): affording birth control (n = 22; 41.5%); affording the visit (n = 17; 32.1%); difficulty making an appointment (n = 16; 30.2%); finding a clinic (n = 15; 28.3%); getting to the appointment (n = 15; 28.3%); and receiving criticism or judgment from the staff/doctors (n = 8; 15.1%). Of the participants who experienced barriers to SRH care, 73.6% reported interest in receiving information about birth control and receiving birth control in the ED if it were available (n = 39). Participants who experienced barriers to SRH services reported higher interest in receiving information and birth control in the ED (74%, n = 39) compared to those who had not experienced barriers (53%, n = 240); (P = 0.004, 95% confidence interval, 1.30–4.66).

In a post hoc fashion we compared interest in ED contraception initiation between participants who were high risk for unintended pregnancy according to the CDC definition^3^ to those who were not in a high-risk group. We found increased rates of acceptability in participants who were 18–24 years of age (n = 95, 68.9%) compared to >24 years of age (n = 221, 60.2%), non-Hispanic Black (n = 153, 63.7%) compared to non-Hispanic White (n = 146, 54.9%), cohabitating but never married (n = 17, 73.9%) compared to any other relationship status (n = 264, 54.8%), and did not complete high school (n = 46, 58.3%) compared to high school diploma/General Education Development or above (n = 237, 55.6%). None of these factors reached statistical significance at a level of *P* = 0.05.

## DISCUSSION

Among the many factors that determine the feasibility of a study, two important elements are that the intervention is both needed and wanted (acceptable) by the target population.^22^ In this survey study of women presenting to the ED, most (55.2%) of our participants wanted to receive contraception and information about contraception in the ED and an additional 7.3% wanted information only. To our knowledge, there has only been one study, published in 2005, examining the acceptability of the provision of contraception in the adult ED population.^23^ In this study, contraception provision in the ED was acceptable to 44% of ED patients. Our rate of acceptability was somewhat higher at 55.2%. This may be secondary to the increase in awareness of and access to contraception over the last decade,^24^ specifically since the introduction of the ACA contraception benefit.^9^,^10^

Todd et al found that acceptability was significantly higher in patients who were uninsured, without a primary care provider, were frequent ED utilizers, and were at increased risk of pregnancy.^23^ In participants who were at increased risk of pregnancy,^3^ we found increased rates of acceptability in most categories including those who were 18–24 years of age, non-Hispanic Black, cohabitating but never married, and had not completed high school. We did not collect income information; therefore, we could not compare low to higher income participants. None of these factors reached statistical significance at a level of *P* = 0.05; however, this study was not powered to answer this question. Additionally, patients who experienced barriers to SRH care reported higher interest in receiving information and birth control in the ED compared to those who did not experience these barriers.

A qualitative study by Caldwell et al found that 81% participants were accepting of contraception counseling in the ED. These participants felt that the ED provided an opportunity to address women’s unmet contraception needs, contraception was within the scope of ED practice, and the ED was a convenient setting with competent providers who could deliver contraception counseling. However, the participants who were not accepting of contraception counseling felt that contraception is a sensitive topic, and the ED is an inappropriate setting to receive contraception counseling.^25^ While this study further supports the ED as a setting for contraception services, it highlights the need for patient-centered, targeted approaches to ED-based contraception services. Future research should explore these factors further.

Our data suggest that ED-based contraception was both wanted and needed. Of participants who were able to but did not want to get pregnant in the next year, 32.6% of them were not using any form of contraception, with another 26.5% relying on condoms only or the withdrawal method. To reduce unintended pregnancy in the US we need to increase access to contraception by identifying alternative settings for its provision^13^,^26^,^27^ because the traditional settings are insufficient to meet the needs of the most vulnerable populations. The need identified by this study supports the notion that the ED may be an important setting to reach some of our patients who are at high risk for unintended pregnancy and its complications.

While our study showed that acceptability of contraception was high in the ED patients we sampled, further research needs to be completed. First, a similar multisite study of acceptability should be implemented to increase generalizability of these findings. Additionally, feasibility studies in the areas of insurance coverage, physician knowledge and acceptability, and follow-up structure as well as a pilot study should be conducted to ensure successful implementation of contraception initiation in the ED.

## LIMITATIONS

Bias may have been introduced into this study as we used a convenience sample rather than a consecutive or random sample. This was because this was an unfunded study. Data was collected by two volunteer RAs, one undergraduate and one medical student. Therefore, data needed to be collected when they were available. While there were no restrictions on when they could collect data, all but two participants were enrolled between 7 am -11 pm. We do not have data on the day of the week data was collected as we did not keep track of dates in order to preserve anonymity and not collect personal health information. Additionally, although we collected data at two large urban EDs, these EDs were located in the same city, limiting generalizability of the results of this study. Another limitation is that we did not keep track of patients who were approached but refused to participate. Therefore, we could not calculate a response rate, and we could not determine whether there was a difference between participants and non-participants. Finally, although insurance status was identified in a prior study as having a significant correlation with acceptability of contraception in the ED,^23^ this survey was not designed to assess influence of insurance status on decision-making; one of the reasons for this was our concern about confounding from financial literacy,^28^ because this was coming from the patient not the chart. We did not have IRB approval to look at the electronic health record. This correlation will be explored in future studies.

## CONCLUSION

The majority of women of childbearing age indicated the desire to access contraception services in the ED setting. This finding suggests favorable patient acceptability for an implementation study of contraception in emergency care.

## Supplementary Information



## Figures and Tables

**Figure:**
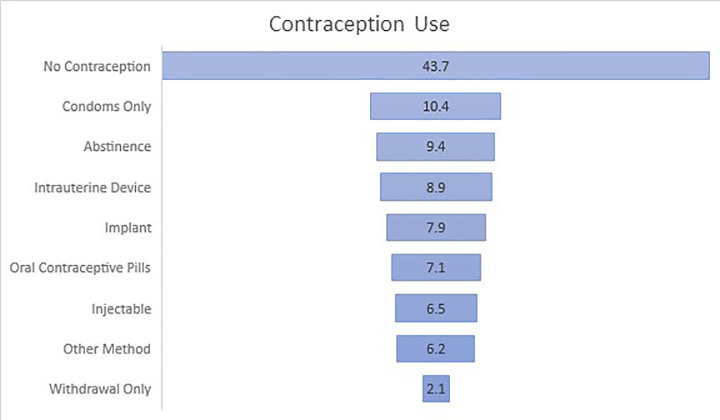
Percentage of contraception use by method in participants with the ability to get pregnant (N = 215). (Participants could choose more than one option; thus, the total is > 100%.)

**Table:** Demographics of female patients who participated in a survey regarding access to sexual and reproductive healthcare.

Demographics		
Age	Range	Mean
		
	18–55	30.7
Race	n	%
		
Black	240	47.5
White	204	40.4
Other	40	7.9
More than one race	9	1.8
Asian	3	0.6
American Indian or Alaskan Native	2	0.4
Missing	7	1.4
Ethnicity		
Not Hispanic or Latinx	425	84.2
Hispanic or Latinx	56	11.1
Missing	24	4.8
Highest level of education		
Some high school	79	15.6
High school/GED	230	45.5
Some college	113	22.4
College	51	10.1
Advanced degree	18	3.6
Trade school	13	2.6
Missing	1	0.2
Relationship status		
Single	276	54.7
Married	97	19.2
Partnered	75	14.9
Cohabitating	23	4.6
Separated	17	3.4
Divorced	16	3.2
Widowed	1	0.2
Desire for pregnancy in the next year		
Yes	71	24.4
No	279	55.2
Can’t get pregnant	123	24.4
Unsure	32	6.3
Site of usual SRH care		
Primary care physician	200	39.6
Outpatient clinic	116	23
Gynecologist	101	20
Nowhere	62	12.3
Emergency department	36	7.2
Planned parenthood	28	5.5
Interest in contraception in the ED		
Information and contraception	279	55.2
No information or contraception	187	37
Information only	37	7.3
Unsure	2	0.4

GED, General Education Development

*SRH*, sexual and reproductive health; *ED*, emergency department.
