# Supplementary material for: Acceptability of Contraceptive Services in the Emergency Department: A Cross-sectional Survey
Source: West J Emerg Med. 2021 May 24;22(3):769–74. doi: 10.5811/westjem.2021.2.49675 (PMC8203030; doi:10.5811/westjem.2021.2.49675)
Supplement: Supplementary file 1 [file wjem-22-769-s001.docx]

*Page 1*

# Supplemental Appendix: Survey

# Demographics and Medical History

Record ID

Time of verbal consent

Age

Hospital of Enrollment Eskenazi

Methodist


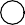

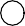


Chief Complaint

Race American Indian or Alaskan Native

Asian


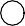

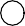

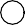

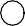

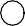

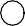

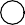


Native Hawaiian or other Pacific Islander Black or African-American

White

More than one race Other

Ethnicity Hispanic or Latino

Not Hispanic or Latino


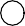

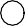


Gravida

Para

Alcohol Use None

A few times per year


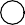

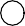

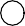

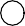

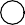

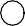


A few times per month Once per week

Several times per week Every day

Drug Use None

Cocaine Crack Heroin Opioid pills


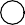

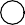

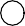

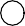

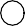

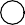

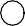

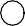


Other opioid injectables Marijuana

Other

Other

Medical History

*Page 2*

Highest level of education completed Some high school High school/GED Some college Trade school College

Advanced degree (MS or higher)


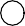

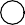

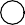

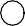

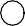

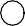


Employment Status Employed full-time

Employed part-time


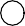

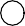

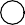

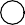

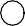


not currently working for pay Not currently employed, retired

Not currently employed, not retired

Student Status Full-time student

Part-time student Not a student


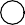

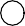

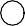


Relationship Status Single

Partner Cohabitating Married Separated Divorced Widowed


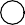

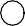

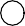

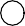

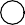

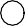

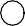


# Survey

*Page 1*

Record ID

Are you currently now or have you ever been sexually Yes active? No

Other


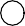

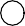

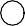


Other

Do you ever trade sex for money, drugs, housing, or Yes

anything else? No


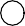

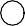

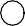


Prefer not to answer

Would you like to become pregnant in the next year? Yes No

Unsure


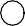

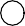

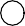

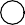


I can't get pregnant

Reason I don't have sex with men

I have had a hysterectomy


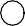

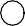

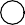

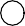


I have gone through menopause

I have had permanent sterilization (aka "tubes tied")


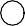
 Other

Other

Have you started trying or will you start trying Yes

soon? No


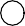

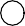


Are you currently using anything to prevent Yes

pregnancy? No


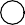

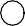

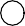


Unsure

What are you using to prevent pregnancy?

Intrauterine device (Mirena, Paragard, other) Contraceptive implant (Implanon, Nexplanon) Injectable birth control (aka Depo, DMPA, "the shot")

Birth control pills Birth control patch

Birth control vaginal ring (aka Nuvaring) Condoms

Withdrawal (aka "the pull-out method") Natural family planning (avoiding sex on fertile days, aka "fertility awareness")

Abstinence (not having sex) Other

Why are you not currently using birth control?

I don't want to

I have a medical condition that prevents me from using birth control

I haven't found a method that works for me I can't afford it

I don't think I'm at risk of getting pregnant

I don't have a doctor or other place to get it Other

In the past 6 months have you used condoms: Never


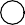

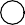

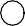


Every time I have sex

Sometimes, but not every time I have sex

Do you use condoms to prevent STIs or to prevent Prevent STIs

pregnancy? Prevent pregnancy


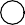

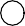

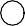

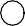


Both Other

Are you interested in hearing more information about Yes

birth control or switching to a different method? No Unsure Other


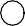

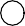

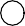

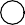


Which birth control method do you feel would best Permanent sterilization (aka "tubes tied") suit your lifestyle? Intrauterine device (Mirena, Paragard)

Contraceptive implant (Implanon, Nexplanon) Injectable birth control (aka Depo, DMPA, "the shot")


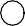

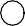

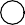

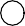


Birth control pills Birth control patch


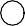

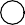

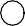

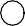

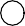

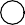


Birth control vaginal ring (aka Nuvaring) Condoms

Withdrawal (aka "the pull-out method") Natural family planning (avoiding sex on fertile days of the cycle, aka "fertility awareness") Abstinence (not having sex)


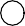

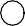


Other

Where do you currently seek care for things like birth control, STIs, pap smears, or other GYN health issues?

Primary care physician Gynecologist

Eskenazi Emergency Department Methodist Emergency Department Other Emergency Department Planned Parenthood

Eskenazi outpatient clinic Other outpatient clinic Nowhere

Which one?

Have you had any difficulty getting care for things Yes

like birth control, STIs, pap smears, or other GYN No


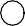

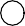

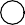

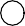


health issues? Unsure

Other

Other

What difficulties have you had? Check all that apply:

Difficulty finding a clinic to go to Difficulty making an appointment Difficulty getting to an appointment Difficulty affording the visit

Difficulty affording birth control, medications, etc.

Receiving criticism or judgment from clinic/staff/doctors/etc.

Other

Other

Would you be interested in receiving information about birth control or getting birth control in the ER if it was available?

Yes, information AND birth control information ONLY

No Unsure Other

Other

Would you be interested in getting a referral to see Yes, both information AND birth control someone in a clinic to get more information about Yes, information ONLY

birth control or to start birth control, if you No


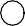

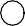

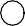

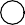

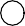


wanted? Unsure

Other

Other
